# Laparoscopic Extended Left Lateral Sectionectomy for Hepatocellular Carcinoma in a Patient with Right-Sided Ligamentum Teres: A Case Report and Literature Review

**DOI:** 10.3390/diagnostics13152529

**Published:** 2023-07-29

**Authors:** Yuki Adachi, Hiroyuki Takahashi, Tomohiro Yamamoto, Masahiro Hagiwara, Koji Imai, Hideki Yokoo

**Affiliations:** Division of Hepato-Biliary-Pancreatic Surgery and Transplant Surgery, Department of Surgery, Asahikawa Medical University, 2-1-1 Midorigaoka Higashi, Asahikawa 078-8510, Japan; a-yuki@asahikawa-med.ac.jp (Y.A.); takahiro@asahikawa-med.ac.jp (H.T.); kyokui100053@gmail.com (T.Y.); fudoushin_hms@yahoo.co.jp (M.H.); kimai@asahikawa-med.ac.jp (K.I.)

**Keywords:** right-sided ligamentum teres, three-dimensional computed tomography, intraoperative ultrasonography, laparoscopic hepatectomy, expanded left lateral sectionectomy

## Abstract

Right-sided ligamentum teres (RSLT) is a rare anatomic variant in which the fetal umbilical vein connects to the right portal vein. Patients with RSLT frequently have hepatic vasculature and bile duct anomalies, which increase the risk of complications with hepatectomy. Most patients with RSLT undergo open hepatectomy. Herein, we describe a patient with RSLT and hepatocellular carcinoma who underwent laparoscopic hepatectomy. The patient was a 69-year-old man with hepatocellular carcinoma located in the left liver based on computed tomography (CT) and magnetic resonance imaging. Imaging also demonstrated RSLT. Three-dimensional CT analysis revealed independent right lateral type anomalies of the portal vein and bile duct. A laparoscopic extended left lateral sectionectomy was performed after careful surgical planning. Ultrasonography was used frequently during surgery to avoid damaging the right hepatic vasculature. The left lateral and partial left median sections were removed as planned. The patient’s postoperative recovery was uneventful. Avoiding injury to the right hepatic vasculature is essential when performing left lobectomy, including left lateral sectionectomy, in patients with RSLT. Laparoscopic hepatectomy can be performed safely in patients with RSLT, provided that careful surgical planning is conducted using preoperative three-dimensional CT analysis and intraoperative ultrasonography.

## 1. Introduction

Right-sided ligamentum teres (RSLT) is a rare anatomical variant in which the fetal umbilical vein is connected to the right paramedian trunk of the portal vein instead of the left portal vein [1]. The reported incidence rates of RSLT range between 0.2% and 1.2% [1,2]. The ligamentum teres is the remnant of the functioning fetal umbilical vein. RSLT is thought to result from obstruction of the left umbilical vein during development, which causes persistence of the right umbilical vein; normally, the right umbilical vein obliterates and the left one persists [3]. In the past, many cases of RSLT were reported as a left-sided gallbladder because the ligamentum teres and gallbladder often assumed the opposite position to normal [1,2]. However, a left-sided gallbladder associated with RSLT is actually an abnormality of the position of the ligamentum teres and should be distinguished from a “true” left-sided gallbladder, which is an abnormality of the position of the gallbladder itself [2,4]. Liver resection in patients with RSLT should be performed cautiously because this variant is associated with a high frequency of portal vein, hepatic vein, and bile duct abnormalities [5]. For this reason, most liver tumor patients with RSLT undergo open hepatectomy [6]. Herein, we report a patient with RSLT who successfully underwent laparoscopic extended lateral sectionectomy for the resection of hepatocellular carcinoma.

## 2. Case Report

**Figure 1:**
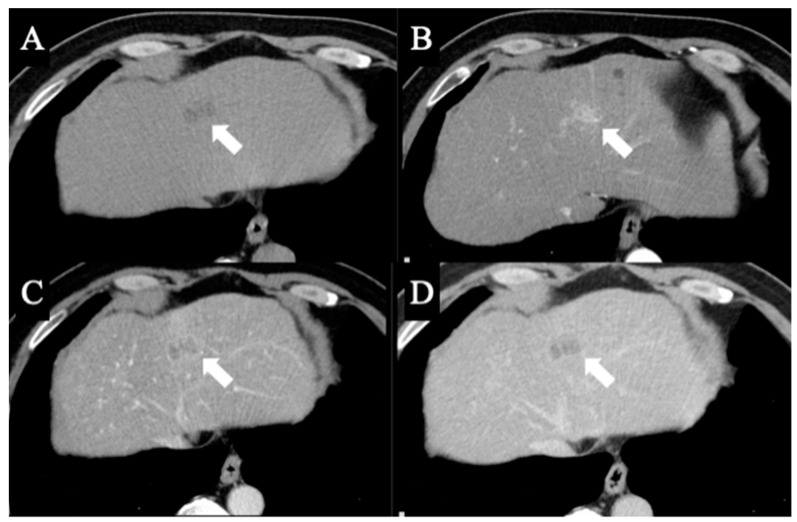
(**A**) Computed tomography showing a 2.4 × 2.0 cm hypodense mass in the left liver. In arterial (**B**), portal (**C**), and delayed (**D**) phase contrast-enhanced images, the mass exhibited early arterial enhancement and washout. The arrows indicate the location of the tumor in each phase.

**Figure 2:**
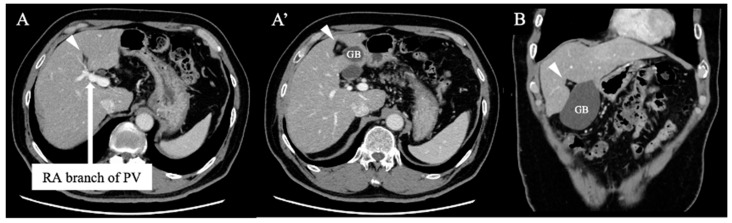
Axial (**A**,**A’**) and coronal (**B**) contrast-enhanced computed tomography demonstrated that the ligamentum teres (arrowhead) was connected to the right anterior (RA) branch of the portal vein (PV) and the gallbladder (GB) was located to its left, indicating right-sided ligamentum teres.

**Figure 3:**
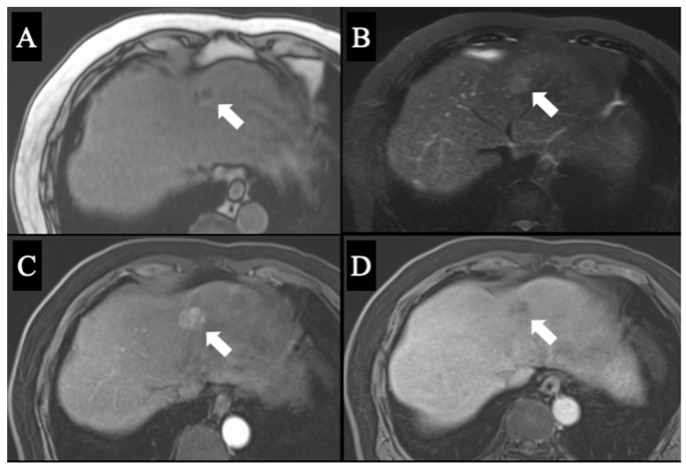
Magnetic resonance imaging with gadolinium ethoxybenzyl diethylenetriamine penta-acetic acid contrast agent revealed a mass that exhibited low signal intensity in T1-weighted images (**A**) and high signal intensity in T2-weighted (**B**) and diffusion-weighted images (**C**). Contrasted images showed a perfusion defect in the mass in the liver phase (**D**). The arrows indicate the location of the tumor in each condition.

**Figure 4:**
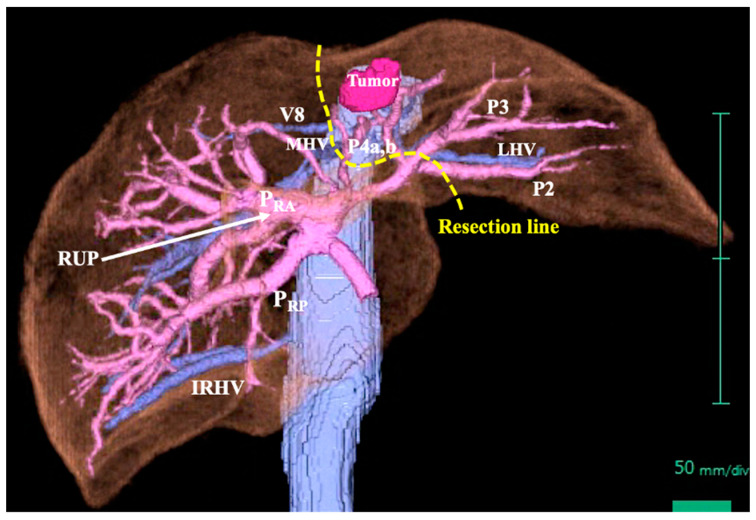
Three-dimensional computed tomography. The tumor (2.4 × 2.0 cm) was in the left lobe of the liver (segment 2/3/4b). The portal vein had a right posterior branch (P_RP_), right anterior branch (P_RA_), and left branch. The PRP branched independently. The left branch branched into a lateral superior branch (P2) and a lateral inferior branch (P3) after two medial branches (P4a,b) separated. The PRA was connected to the right umbilical portion (RUP). A right inferior hepatic vein (IRHV) and a hepatic vein branch of an anterior superior segment (V8) were present. The middle hepatic vein (MHV) and left hepatic vein (LHV) were normal. The planned resection line is indicated by the yellow dotted line.

**Figure 5:**
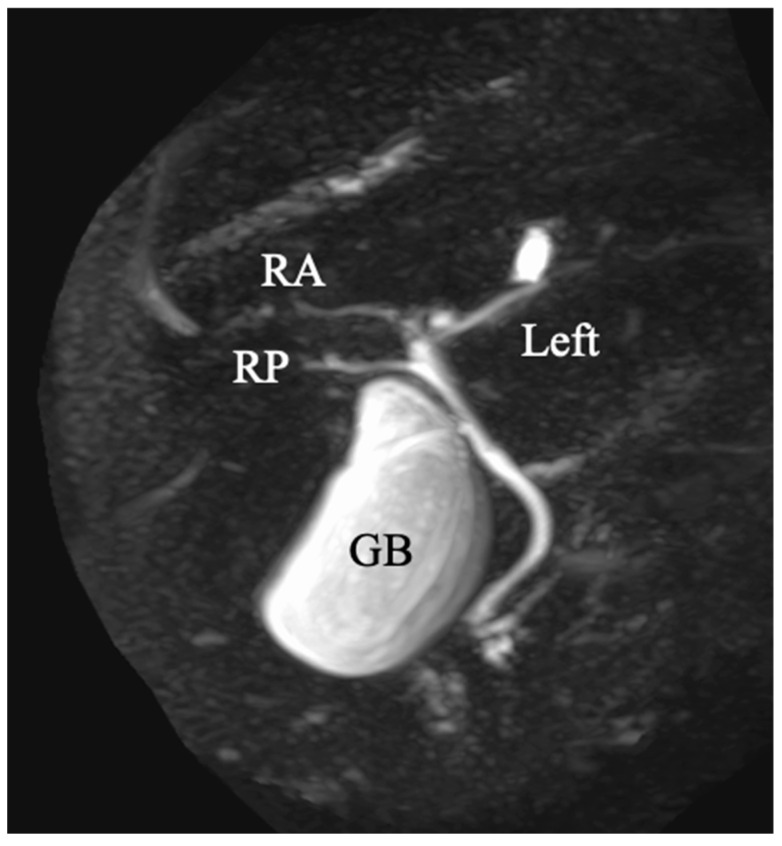
Magnetic resonance cholangiopancreatography revealed that the bile duct system was trifurcated into right posterior (RP), right anterior (RA), and left branches. GB indicates the gallbladder.

A 69-year-old man was referred for an evaluation of a liver tumor detected on abdominal ultrasonography performed for a nonalcoholic steatohepatitis follow-up. Blood testing showed elevations in concentrations of aspartate transaminase (56 U/L), alanine transaminase (51 U/L), and protein induced by vitamin K absence-II (224 mAU/mL). Other tumor markers and hepatitis virus markers were negative. The indocyanine green retention rate at 15 min was 15.2% and the Child–Pugh score was 5 points (class A), suggesting good hepatic function. Computed tomography (CT) showed a 2.4 × 2.0 cm hypodense mass in the left lobe of the liver (segment 2/3/4b) which exhibited early arterial enhancement and washout (Figure 1). Hepatocellular carcinoma was highly suspected. In addition, the ligamentum teres was connected to the right anterior branch of the portal vein, and the gallbladder was located to its left, indicating RSLT (Figure 2). On magnetic resonance imaging, the mass had low signal intensity on T1-weighted images and high signal intensity on T2-weighted and diffusion-weighted images; contrasted imaging using gadolinium ethoxybenzyl diethylenetriamine penta-acetic acid revealed a perfusion defect in the mass in the liver phase (Figure 3). Three-dimensional CT (Synapse Vincent^®^ Fujifilm Medical, Tokyo, Japan) demonstrated the right posterior, right anterior, and left branches of the portal vein. The left branch branched into a lateral superior branch (P2) and a lateral inferior branch (P3) after two medial branches (P4a,b) separated. A right inferior hepatic vein and a hepatic vein of an anterior superior segment (V8) were present (Figure 4). Magnetic resonance cholangiopancreatography revealed that the bile duct system was trifurcated into the right posterior, right anterior branch, and left branches (Figure 5). After the pertinent anatomy was clarified and the preoperative evaluation was completed, we elected to perform laparoscopic extended lateral sectionectomy.

**Figure 6:**
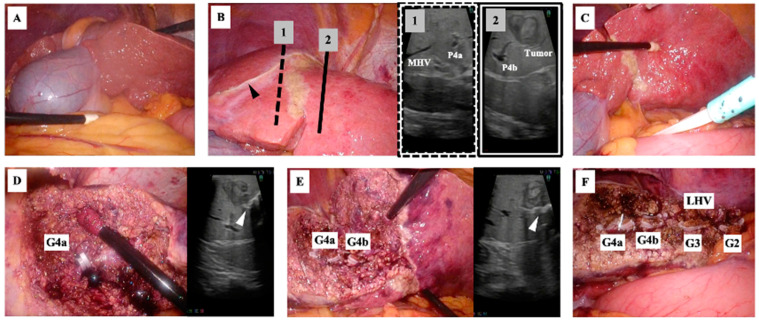
Intraoperative photography. (**A**) The ligamentum teres was positioned on the right and the gallbladder is on the left. (**B**) After the locations of the tumor and middle hepatic vein (MHV) were confirmed using ultrasonography, the resection line was planned to avoid damaging the MHV. The hepatic attachments of the falciform ligament (black arrowhead) were located more to the right than normal. P4a and P4b indicate the medial inferior and medial superior branches of the portal vein, respectively. (**C**) The planned resection line on the undersurface of the liver was marked. (**D**,**E**) The extent and depth of resection (white arrowhead) were confirmed using ultrasonography. The medial inferior (G4a) and medial superior branches of Glisson (G4b) were clipped and cut. (**F**) Resection of the liver parenchyma advanced in a left dorsal direction, clipping and cutting the lateral superior (G2) and lateral inferior branches of Glisson (G3) and the left hepatic vein (LHV). Finally, the left lateral section, including the partial left median section, was resected.

Surgery was performed with the patient in the supine position using a 2K high-definition/three-dimensional monitor. Five laparoscopy ports were placed: a 12 mm camera port at the umbilicus, 12 mm ports below the xiphoid process and the right rib arch, and 5 mm ports outside the right and left rib arches. After confirming the RSLT and the location of the gallbladder (Figure 6A), the falciform ligament and left triangular ligament were dissected. The tumor was then located using ultrasonography. To determine the extent of resection while avoiding damage to the right-sided vasculature, the head side was marked at the boundary between the segment 4b and the right anterior section (Figure 6B), and the caudal side was marked at the line where the segment 4a remained (Figure 6C). The liver resection was performed using a cavitational ultrasonic surgical aspirator (CUSA^®^ Excel; Integra, Princeton, NJ, USA) and ultrasonic scalpel (Harmonic^®^ HD 1000i shears; Ethicon, Raritan, NJ, USA). The medial inferior, medial superior, lateral inferior, and lateral superior branches of Glisson and left hepatic vein were individually double-clipped using a polymer locking ligation system (Hem-o-lok^®^; Teleflex, Wayne, PA, USA) and resected. Thin vein branches were clipped using a metallic clip applier (Endo Clip^®^; Medtronic, Dublin, Ireland) and cut. Finally, the left lateral section, including the partial left median section, was resected (Figure 6D–F). The operation time was 7 h and 35 min. The blood loss volume was 109 mL. The patient’s postoperative recovery was uneventful and he was discharged on postoperative day 7. The final pathological diagnosis was well-differentiated hepatocellular carcinoma.

## 3. Discussion

To the best of our knowledge, laparoscopic sectionectomy for a malignant liver tumor in a patient with RSLT has been reported only once previously [6]. Most patients with RSLT are treated using open surgery because of the high frequency of vascular and bile duct abnormalities [5]. However, the laparoscopic approach can be safe and effective with adequate preoperative and intraoperative imaging evaluations.

Our patient exhibited an independent posterior branch of the portal venous system, which is present in more than half of patients with RSLT [1,2,5]. Shindoh et al. classified RSLT into three types based on the portal branching pattern: bifurcation, trifurcation, and independent right lateral (posterior) type [1]. In contrast, Terasaki et al. classified patients into four types: one bilateral ligamentum teres group and three RSLT groups (bifurcation, trifurcation, and independent posterior branch types) [5,7]. The newer Terasaki system may become the standard in the future. Regarding hepatic veins in patients with RSLT, a thick vein running along the ventral–dorsal segment border of the anterior sector and a developed V8 are likely to be present [1,5]. Our patient exhibited a developed V8. Because the middle hepatic vein (MHV) in patients with RSLT tends to be atrophic and displaced medially, the V8 may be misidentified as the MHV and should be treated with caution [5]. As for the hepatic artery, most bifurcate and branch normally in patients with an RSLT, as in our patient [1,8].

The bile duct system frequently does not bifurcate normally in patients with RSLT. Nishitai et al. classified the biliary architecture in such patients into four types: the symmetrical type, in which the right umbilical portion (RUP) is the watershed between the right and left branches of the bile duct; the independent right lateral type, in which the right posterior branch is independent and the right anterior sector branch comes around from the left side of the RUP; and the total left and total right types, in which the entire liver is drained only by the bile duct coming around the RUP from one direction [8]. In their study, half were symmetrical and 28% were the independent right lateral type, as in our patient.

The presence of these vascular and bile duct system abnormalities can lead to unexpected complications [6,8]. In our patient, both the portal vein and the bile duct had a bifurcation type in which the right posterior branch independently diverged; this branch then diverged into the right anterior and left branches. In such cases, caution is required when performing a left lobectomy, including left lateral sectionectomy, because an incision that is too deep or more right-sided than planned may result in injury to the right anterior branch. In addition, the ligamentum teres and the falciform ligament do not represent landmarks for the P2 and P3 origins in patients with LSRT [6]. Furthermore, the misidentification of a developed V8 as MHV may lead to a situation in which a planned left lobectomy must be converted to a left trisectionectomy. These possibilities were all considered in our case. However, the points to keep in mind will vary depending on the particular combination of anatomical anomalies and the type of surgery planned.

Fifteen cases of hepatectomy for malignant tumors in patients with RSLT have been reported in the English literature: thirteen open and two laparoscopic (Table 1) [2,6,7,9,10,11,12,13,14,15,16,17]. Three-dimensional liver anatomy simulation systems using CT images allow a detailed and easy evaluation of tumor location and vasculature. Several reports have shown that such an evaluation is quite useful in RSLT cases characterized by many anatomical abnormalities [6,15,16]. Compared with open surgery, many operations are more difficult when performed laparoscopically because of instrument angle limitations and the inability to directly observe the intraperitoneal cavity. Therefore, a careful preoperative evaluation of tumor location, progression, and anatomy is necessary. With a thorough evaluation and frequent use of ultrasonography during surgery, laparoscopic hepatectomy can be performed safely in many patients.

## 4. Conclusions

RSLT is frequently associated with abnormalities of the liver vasculature and bile duct system. These abnormalities can lead to complications when performing hepatectomy. With left lobectomy, including left lateral sectionectomy, it is important to avoid injury to the right liver vasculature. Laparoscopic hepatectomy can be performed safely in patients with RSLT, provided that careful surgical planning is conducted using preoperative three-dimensional CT analysis and intraoperative ultrasonography.

## Figures and Tables

**Table 1:** Previously reported cases of hepatectomy for malignant tumors in patients with right-sided ligamentum teres.

No	Author	Year	Age	Sex	Disease	Operation	Open/Lap	Complication	Portal Vein Branching Type
1	Uesaka et al. [9]	1995	53	M	Liver metastasis of CC	Right hemihepatectomy	Open	-	Independent right lateral
2	Nagai et al. [2]	1997	67	M	HCC	Segmentectomy 8, and partial resection of segment 1	Open	-	Independent right lateral
3	Nagai et al. [2]		67	M	Hilar CC	HPD	Open	-	Independent right lateral
4	Kaneoka et al. [10]	2000	53	M	Hilar CC	Extended left hemihepatectomy with extrahepatic bile duct resection	Open	-	Independent right lateral
5	Kaneoka et al. [10]		61	M	Extrahepatic CC	HPD	Open	-	Independent right lateral
6	Tashiro et al. [11]	2003	53	M	HCC	Partial hepatectomy	Open	-	Independent right lateral
7	Sakaguchi et al. [12]	2011	76	M	CRLM	Extended right posterior sectionectomy	Open	-	Independent right lateral
8	Abe et al. [13]	2012	70	F	CRLM	Right hemihepatectomy	Open	-	Bifurcation
9	Almodhaiberi et al. [14]	2015	67	M	Hilar CC	Extended left lateral sectionectomy with extrahepatic bile duct resection	Open	-	Trifurcation
10	Ome et al. [15]	2016	70	M	ICC	Right anterior sectionectomy	Open	-	Trifurcation
11	Ome et al. [15]		70	F	Hilar CC	Left trisectionectomy with extrahepatic bile duct resection	Open	-	Trifurcation
12	Hai et al. [16]	2017	78	M	Hilar CC	Extended left hemihepatectomy	Open	Bile leakage	Independent right lateral
13	Goto et al. [17]	2018	70s	M	GC	Right hemihepatectomy	Open	-	other
14	Terasaki et al. [6]	2019	63	M	CRLM	Left lateral sectionectomy	Lap	-	Bifurcation
15	Terasaki et al. [8]	2020	72	M	Hilar CC	HDP	Open	-	Bilateral
16	Our case	2021	69	M	HCC	Extended left lateral sectionectomy	Lap	-	Independent right lateral

RSLT, right-sided ligamentum teres; CC, cholangiocarcinoma; HCC, hepatocellular carcinoma; CRLM, colorectal liver metastasis; HPD, hepatopancreatoduodenectomy; Lap, laparoscopic surgery; Open, laparotomy.

## Data Availability

The original data presented in the study are included in the article. Further inquiries can be directed to hidekiyokoo@asahikawa-med.ac.jp.
